# Circulating Endothelial Nitric Oxide Synthase on the First Postpartum Day: An Exploratory Cross-Sectional Study of Its Association with Maternal Age, Redox-Relevant Haematological and Lipid Markers, and Postnatal Depression Screening

**DOI:** 10.3390/antiox15091176

**Published:** 2026-09-16

**Authors:** Cristina Dragomir, Mihai Mitulețu, Ioana Muntean, Călin Muntean, Peter Seropian, Ionuț-Marcel Cobec, Roxana Popescu

**Affiliations:** 1 Doctoral School of Medicine, “Victor Babeș” University of Medicine and Pharmacy, Eftimie Murgu Square, No. 2, 300041 Timisoara, Romania; cristina.dragomir@umft.ro; 2 ANAPATMOL Research Center, “Victor Babeș” University of Medicine and Pharmacy, Eftimie Murgu Square, No. 2, 300041 Timisoara, Romania; muntean.ioana@umft.ro (I.M.); popescu.roxana@umft.ro (R.P.); 3 Department II Microscopic Morphology, Cell and Molecular Biology, “Victor Babeș” University of Medicine and Pharmacy, Eftimie Murgu Square, No. 2, 300041 Timisoara, Romania; 4 Department of Informatics and Medical Biostatistics, “Victor Babeș” University of Medicine and Pharmacy, Eftimie Murgu Square, No. 2, 300041 Timisoara, Romania; cmuntean@umft.ro; 5 Clinic of Obstetrics and Gynecology, Klinikum Freudenstadt, 72250 Freudenstadt, Germany; peter.seropian@klf-net.de; 6 Department of Obstetrics and Gynecology, Faculty of Medicine, Medical Center-University of Freiburg, 79106 Freiburg, Germany

**Keywords:** endothelial nitric oxide synthase, eNOS, NOS3, nitric oxide, oxidative stress, postpartum, maternal age, endothelial dysfunction, postnatal depression, Edinburgh Postnatal Depression Scale

## Abstract

(1) Background: Endothelial nitric oxide synthase (eNOS/NOS3), the main source of vascular nitric oxide (NO), generates superoxide instead of NO when oxidative stress uncouples it. Circulating eNOS in the early puerperium is uncharacterised. (2) Methods:In a single-centre cross-sectional study (Timișoara, Romania, July 2022), 40 mother–newborn dyads were evaluated. Maternal serum eNOS protein was quantified by ELISA on postpartum day 1, alongside pre-delivery laboratory data and Edinburgh Postnatal Depression Scale (EPDS) screening. Analyses (Spearman correlation, linear regression, prevalence odds) were unadjusted for multiplicity; only correlations of |ρ| ≥ 0.44 were detectable at 80% power. (3) Results:Median serum eNOS was 8.997 ng/mL (interquartile range 4.091–27.985). eNOS correlated inversely with total and LDL cholesterol (ρ = −0.368, *p* = 0.027; ρ = −0.412, *p* = 0.013) and positively with erythrocyte count (ρ = 0.326, *p* = 0.046). The association with maternal age was similar in magnitude but non-significant (ρ = −0.303, *p* = 0.061). Multivariable models were not significant, and eNOS was unrelated to EPDS score (ρ = 0.111, *p* = 0.512). (4) Conclusions:Lower circulating eNOS protein, not enzyme activity, accompanied a less favourable lipid profile, but not depressive symptoms. These single-centre Romanian findings are hypothesis-generating and should not be generalised.

## 1. Introduction

The postnatal period, from delivery to 42 days, is a physiologically demanding transition for mothers and newborns, and maternal and neonatal morbidity and mortality remain unacceptably high; worldwide, a woman dies approximately every two minutes from largely preventable and treatable complications of pregnancy or childbirth [1,2]. This interval is characterised by rapid cardiovascular remodelling, involution of the high-flow uteroplacental circulation, mobilisation of lipid and iron stores, and abrupt endocrine changes, all of which place substantial demand on the vascular endothelium and its redox balance [3,4].

The calcium–calmodulin-dependent flavoprotein homodimer enzyme (~133 kDa) endothelial nitric oxide synthase (eNOS) is encoded by the NOS3 gene on chromosome 7q36.1 and produces nitric oxide (NO) from L-arginine [5,6,7,8,9]. Three variants of NOS have been described: neuronal—nNOS (NOS1) and endothelial—eNOS (NOS3), which are always expressed, and inducible—iNOS (NOS2), an isoform that responds to pro-inflammatory stimuli. NO can also be produced through the NOS-independent nitrate–nitrite–NO pathway [5,10,11,12,13].

Although expressed predominantly in vascular endothelial cells, eNOS is also present in platelets, placenta, perivascular adipose tissue, erythrocytes, and other tissues, and its subcellular localisation (plasma-membrane caveolae, Golgi, cytoskeleton) is dynamically regulated by shear stress, bradykinin, oestrogen, and vascular endothelial growth factor [6,8,14]. eNOS-derived NO activates soluble guanylate cyclase in vascular smooth muscle, raising cyclic GMP to produce vasodilation; it also inhibits platelet aggregation and leukocyte adhesion [11,15]. The discovery of endothelium-derived NO as a cardiovascular signalling molecule was recognised by the 1998 Nobel Prize in Physiology or Medicine awarded to Furchgott, Ignarro and Murad [11,16].

From an antioxidant and redox-biology perspective, eNOS is a paradigmatic redox switch. When its cofactor tetrahydrobiopterin (BH4) is oxidised or L-arginine is limiting, eNOS “uncouples” and generates superoxide (O2•−) rather than NO; the resulting NO/O2•− imbalance forms peroxynitrite, further oxidises BH4, and propagates a self-amplifying cycle of oxidative stress and endothelial dysfunction [17,18,19,20]. Cardiovascular risk factors that intensify oxidative stress, among them advancing age, dyslipidaemia, hypertension, diabetes, and smoking, promote eNOS uncoupling and reduce NO bioavailability. Several of these factors are prevalent during pregnancy and the puerperium [18,19]. Conversely, antioxidant strategies (e.g., vitamins C and E, the glutathione precursor N-acetylcysteine) and dietary nitrate can preserve NO bioavailability and endothelial function [19,21,22]. This redox dependence places eNOS squarely within the scope of maternal oxidative-stress biology.

In reproduction, eNOS-derived NO participates in follicular development, ovulation, implantation, trophoblast invasion, maintenance of uteroplacental perfusion and parturition; impaired NO production has been linked to defective implantation, miscarriage and infertility [23,24]. In hypertensive disorders of pregnancy, placental oxidative stress inhibits and uncouples eNOS, and reduced NO bioavailability is a central mechanism of the endothelial dysfunction underlying pre-eclampsia [25,26,27,28]. Circulating eNOS has been reported to be lower in non-pregnant women and in hypertensive disorders of pregnancy than in healthy pregnancy, and to be associated with maternal haemoglobin and birth weight [25,26]. In anewborn, NO governs the pulmonary and fetoplacental circulation and is used therapeutically as inhaled NO, while nitrite in colostrum supplies NO during the early neonatal period [29,30,31,32].

eNOS is also relevant to maternal mental health. Peripartum depression, and within it postnatal depression (PPD), affects roughly 10–20% of mothers and up to half of cases remain undiagnosed; it impairs maternal functioning and mother–infant bonding and, when untreated, carries substantial long-term risk [33,34,35,36,37]. Because NO functions as a neurovascular signalling molecule, and because reduced platelet NOS activity and lower plasma NO metabolites have been reported in major depressive disorders, while eNOS-deficient animals develop depression-like behaviour [38,39], whether circulating eNOS relates to early depressive symptoms in the puerperium is a biologically plausible but untested question.

Despite this breadth of evidence, circulating eNOS in the immediate puerperium—the very window in which maternal and neonatal physiology adapt most abruptly—has not been characterised, nor have its relationships with routine, redox-relevant clinical markers or with early depression screening. We therefore conducted an exploratory, single-centre cross-sectional study with the following objectives: (a) to quantify maternal serum eNOS on the first postpartum day; (b) to examine its associations with pre-delivery haematological, inflammatory and lipid markers; (c) to test whether eNOS relates to the intensity of PPD symptoms screened with the Edinburgh Postnatal Depression Scale (EPDS); and (d) to explore associations between maternal and neonatal parameters. The primary objective of the study corresponds to objectives a–b: to quantify circulating (serum) eNOS on the first postpartum day and to estimate its association with maternal age and with routine, redox-relevant haematological and lipid markers. Objectives (c) and (d), the relation of eNOS to EPDS-screened depressive symptoms and the maternal–neonatal associations, were secondary. Consistent with this exploratory design, rank-based (Spearman) bivariate correlations were pre-specified as the primary analysis addressing these objectives, with linear regression models and prevalence-odds estimates serving as complementary, hypothesis-generating analyses (Section 2.7). Given the redox nature of eNOS, the analysis of the results will be done in the context of maternal oxidative stress.

## 2. Materials and Methods

### 2.1. Study Design and Setting

This single-centre cross-sectional observational study was carried out in July 2022 at the “Pius Brînzeu” County Emergency Clinical Hospital (Obstetrics–Gynecology and Neonatology clinics) in Timișoara, Romania. The report follows the Strengthening the Reporting of Observational Studies in Epidemiology (STROBE) recommendations for cross-sectional studies (checklist provided as Supplementary Material). Mother–newborn dyads hospitalised in the maternity unit around the time of delivery were consecutively invited to participate.

### 2.2. Participants: Eligibility Criteria

Inclusion criteria (all required):

- Singleton pregnancy with a live birth managed at the study hospital;
- Maternal age ≥ 18 years;
- Written informed consent provided by the mother for herself and her newborn;
- Completion of the EPDS depression screening questionnaire in the early puerperium;
- Availability of the pre-delivery laboratory panel and a postpartum day-1 blood sample.

Exclusion criteria:

- Failure to meet any inclusion criterion;
- Multiple pregnancies;
- Incomplete consent, questionnaire, or laboratory data;
- Haemolysed or insufficient serum sample precluding valid eNOS quantification.

**Figure 1:**
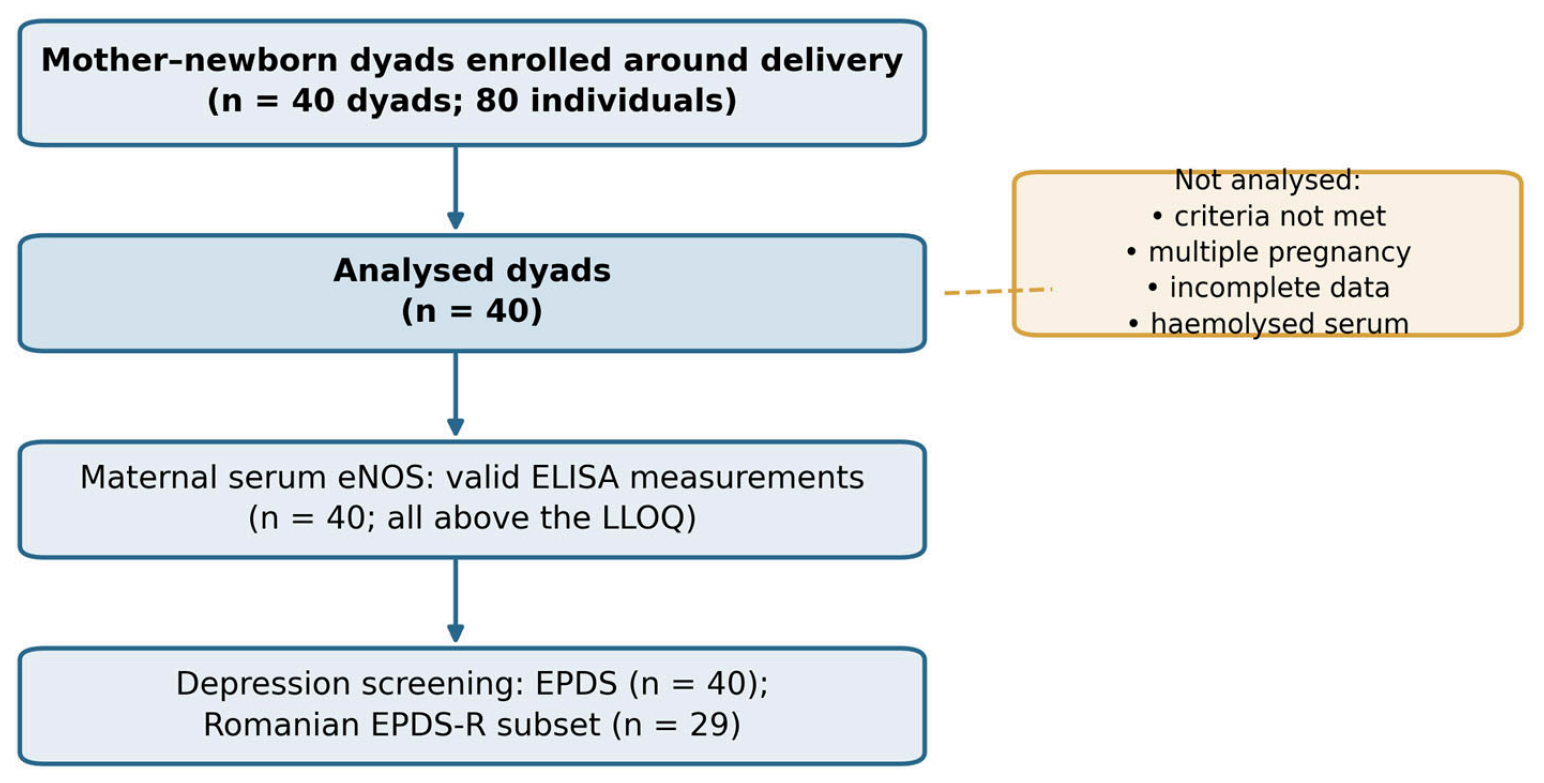
STROBE participantflow diagram of mother–newborn dyad selection (*n* = 40 analysed dyads; valid maternal serum eNOS, *n* = 40; Romanian-language EPDS-R subset, *n* = 29).

Of the dyads screened, 80 individuals (40 mother–newborn dyads)met all criteria and were analysed. Because a small number of samples or questionnaire items were incomplete, the analysable *n* varies by variable and is reported explicitly for every analysis (valid maternal eNOS measurements, *n* = 40; EPDS Romanian-language re-administration, *n* = 29). The participant flow is shown in Figure 1.

### 2.3. Blood Sampling and Pre-Analytical Handling

Maternal venous blood was collected on the morning of the first postpartum day, after an overnight fast, into serum tubes without anticoagulant (BD vacutainer Clot activater tube, BD-Plymouth, Plymouth, UK). Whole blood was allowed to clot at room temperature for 30 min and then centrifuged at 2000× *g* for 10 min at 4 °C in a refrigerated centrifuge (centrifuge model SL 16R, Thermo Fisher Scientific, Osterode am Harz, Germany). Serum was aliquoted and stored at −80 °C until analysis, avoiding repeated freeze–thaw cycles. Routine pre-delivery investigations (complete blood count with erythrocyte and platelet indices, C-reactive protein [CRP], lipid profile, creatinine, electrolytes, and urinalysis) were obtained from the clinical laboratory record (complete blood count on a (CELLTAC G+ 9200) automated haematology analyser (Nihon Kohden, Tokyo, Japan); biochemistry and CRP on a Atellica Solution—CH1 analyser, Siemens Healthcare Diagnostics Inc., Tarrytown, NY, USA); corresponding neonatal day-1 parameters were recorded.

### 2.4. Quantification of Serum eNOS by ELISA

The determination of the eNOS/NOS3 level in human serum was done using a sandwich immunoassay (ELISA) (Invitrogen/Thermo Fisher Scientific, Life Technologies Corporation, Frederick, MD, USA, catalogue no. EH169RB) strictly according to the manufacturer’s protocol. All samples and standards were assayed in triplicate. A calibration curve (optical density versus standard concentration) was constructed for each plate. Optical density was read at 450 nm on a Varioskan LUX Multimode Microplate Reader (Thermo Fisher Scientific, Singapore), and concentrations were interpolated from each plate-specific standard curve using a four-parameter logistic (4PL) non-linear regression model in GraphPad Prism v10.2.2 (GraphPad Software, Boston, MA, USA). GraphPad Prism was used because it implements the 4PL sigmoidal fit recommended for sandwich immunoassays, in which the optical density–concentration relationship is non-linear across the assay range, so the 4PL model provides more accurate interpolation than linear calibration [40].

The assay range was 0.4–100 ng/mL, and intra- and inter-assay coefficients of variation (CV) were below 10%. In line with clinical-chemistry recommendations for reporting the detection capability of quantitative assays [41], the limit of detection (LOD; analytical sensitivity), established in the manufacturer’s validation, was 0.4 ng/mL, and the lowest calibration standard (0.4 ng/mL) was adopted as the lower limit of quantification (LLOQ). All valid serum eNOS measurements (*n* = 40) were above the LLOQ, the lowest observed concentration (2.537 ng/mL) exceeding it more than six-fold; consequently, no result was censored or excluded as being below the LOQ, and neither the effective sample size nor the statistical power was reduced by assay sensitivity. The LOD and LLOQ used here are those established by the manufacturer; they were not independently re-verified in-house in the serum matrix of this study, and no in-house estimate of the limit of blank, of matrix-specific recovery or of parallelism was generated. The detection capability of the assay in this specific matrix is therefore assumed rather than demonstrated, which is acknowledged as a methodological limitation (Section 4.6). The assay quantifies total immunoreactive eNOS protein: it does not distinguish phosphorylated (e.g., Ser1177) from non-phosphorylated enzyme or dimeric from monomeric forms, and it therefore does not measure eNOS enzymatic activity (Section 4.2 and Section 4.6). Samples exceeding the upper limit of the standard curve (100 ng/mL) were re-analysed after dilution in the kit sample diluent, and the result was multiplied by the dilution factor; this applied to the single highest observation (264.737 ng/mL).

### 2.5. Postnatal Depression Screening

Depressive symptoms were screened in the early puerperium with the 10-item Edinburgh Postnatal Depression Scale (EPDS), a validated screening rather than diagnostic instrument covering the preceding 7 days [42]. Items are scored 0–3 according to symptom intensity (items 3, 5 and 10 are reverse-scored), giving a total of 0–30; any positive score on item 10 was treated as a suicidality flag requiring immediate clinical evaluation [43]. Four bands were used throughout: low probability, score < 9; possible depression, 9–11; fairly high probability, 12–13; and probable depression, ≥14 [42,43]. A Romanian-language version of the scale (EPDS-R) [44] was also administered to a subgroup (*n* = 29) as an internal consistency check, using the same four bands. Two caveats apply to that subgroup and are carried through to the interpretation. First, the Romanian instrument was validated in an antenatal population, so its performance in the immediate puerperium is not established. Second, the subgroup is small, so the EPDS-R results are reported descriptively, alongside the agreement between the two versions, and are not used to reclassify any participant. The screening accuracy of the EPDS in perinatal populations, with and without the self-harm item, has been examined elsewhere [43].

### 2.6. Haematological Indices

Erythrocyte indices, namely mean corpuscular volume (MCV), mean corpuscular haemoglobin (MCH), mean corpuscular haemoglobin concentration (MCHC) and red cell distribution width (RDW-CV), together with platelet indices (mean platelet volume [MPV], platelet distribution width [PDW]) were used to characterise and classify anaemia, computed by standard formulae: MCV = (haematocrit × 10)/red blood cell (RBC) count; MCH = (haemoglobin × 10)/RBC count; MCHC = (haemoglobin/haematocrit) × 100 [45,46,47]. For this analysis, gestational anaemia was defined operationally as haemoglobin < 11.5 g/dL together with haematocrit < 35% [48]. Two features of that rule should be stated plainly. It is conjunctive, whereas current guidance defines anaemia based on haemoglobin and treats haematocrit as an alternative rather than a co-requirement; furthermore, both thresholds lie above the corresponding guideline values. WHO tabulates trimester-specific cut-offs of <110 g/L in the first and third trimesters and <105 g/L in the second [49], while UK haematology guidance uses <110 g/L in the first trimester, <105 g/L in the second and third and <100 g/L postpartum [50]. The operational definition is retained here because it was applied prospectively and determines the classification reported in Section 3.2, but the resulting prevalence is not comparable with guideline-based estimates, and this is carried into the limitations (Section 4.6).

### 2.7. Statistical Analysis

Data were managed in Microsoft Excel (Microsoft 365, version 2026; Microsoft Corporation, Redmond, WA, USA) and analysed in R version 4.5.2 (R Foundation for Statistical Computing, Vienna, Austria), PAST version 4.03 (Natural History Museum, University of Oslo, Oslo, Norway) and MedCalc version 23.6.1 (MedCalc Software Ltd., Ostend, Belgium) [51,52,53,54].Distributional normality was assessed with the Shapiro–Wilk test (test statistic W, where values closer to 1 indicate closer agreement with a normal distribution; the test performs well at the sample sizes studied here); a variable was considered normally distributed at *p* ≥ 0.05. Normally distributed variables are summarised as mean ± standard deviation (SD) and non-normal variables as the median and interquartile range, denoted MED (IQR); the IQR is reported either as the Q1–Q3 interval or as its width “Q3 − Q1”, as specified for each result. Because most variables were non-normally distributed, Spearman’s rank correlation coefficient (ρ), which is robust to non-normality and to extreme values, was pre-specified as the primary measure of bivariate association.

Simple and multiple linear regression were used as complementary, secondary analyses with a distinct purpose: to estimate the direction and magnitude of the linear component of the associations and to evaluate whether they persisted after mutual adjustment. The coefficient of determination (*R*^2^) refers exclusively to the fit of the linear model and is neither derived from, nor numerically interchangeable with, Spearman’s ρ, which quantifies monotonic association on ranked data. Given the small sample and the skewed eNOS distribution with one extreme value, the assumptions of linear regression (linearity, normality of residuals, homoscedasticity) cannot be guaranteed; regression estimates are therefore reported as descriptive, hypothesis-generating complements to the primary rank-based analysis, and their sensitivity to the extreme observation is explicitly acknowledged (Section 3.6 and Section 4.6). Prevalence odds and odds ratios (OR) with 95% confidence intervals (CI) were computed for binary maternal and neonatal outcomes.

The study is explicitly exploratory and generates hypotheses; given the small sample size and the magnitude of the pairwise correlations studied, the *p*-values are two-sided. They were NOT adjusted for multiple comparisons and, as a result, the associations should be interpreted as exploratory and requiring confirmation. A sensitivity/precision analysis showed that, with the 36 to 40 pairs available per comparison, α = 0.05 (two-sided) and 80% power, the minimum detectable correlation was |ρ| ≈ 0.44; the study was therefore adequately powered only for moderate-to-strong associations, and small effects (e.g., ρ ≈ 0.1) could not be reliably detected (posthoc power for ρ = 0.11 was approximately 10%, and the age association at ρ = −0.303 carried only 47% power). Statistical significance was set at *p* < 0.05, with 0.05 ≤ *p* < 0.10 regarded as marginal. Because eNOS was strongly right-skewed with one extreme high value (264.7 ng/mL), the median (IQR) is used as the primary summary; rank-based analyses, which are insensitive to the magnitude of extreme values, constitute the primary correlation method, and the influence of this observation on the secondary linear models is addressed in Section 3.6.

The same reasoning was applied to the regression models, which are secondary throughout. For the simple model of eNOS on maternal age (R^2^ = 0.081, *n* = 39), the corresponding effect size is f^2^ = 0.09 and the posthoc power at α = 0.05 is only 44%, well short of the conventional 80% criterion. For the multivariable models, a sensitivity analysis is more informative than observed power: at α = 0.05, the smallest effect detectable with 80% power is f^2^ = 0.29 (R^2^ ≈ 0.22) for the two-predictor model and f^2^ = 0.34 (R^2^ ≈ 0.26) for the three-predictor model—that is, only large effects in Cohen’s terms. Two consequences follow and are applied consistently in the Results and Discussion. Non-significance of the multivariable models is uninformative and is not read as evidence that the predictors are unrelated to eNOS; and no predictor, maternal age included, is described as an independent predictor of eNOS on the basis of these models.

### 2.8. Ethics

The study was carried out in accordance with the Declaration of Helsinki and with the approval of the Ethics Committee for Scientific Research at Victor Babes University of Medicine and Pharmacy, Timisoara, Romania (protocol code 07, approved 28 February 2020, revised in 2023). Participating mothers signed written consent for themselves and their newborns, also giving permission to use anonymized data for analysis and publication.

### 2.9. Use of Generative AI

Generative AI tools were only used to assist with language editing and formatting of the manuscript; they were not used to generate, analyse, or interpret study data. The authors reviewed and edited all output and take full responsibility for the content.

## 3. Results

### 3.1. Participant Characteristics

Forty mothers were analysed, all aged 40 years or younger (mean age 29.9 years, range 18–39). The recorded age bands were 18–20 years, *n* = 1; 21–30 years, *n* = 20; and 31–40 years, *n* = 18; maternal age was not recorded for one participant. Sixty percent (*n* = 24) came from an urban environment and 40% (*n* = 16) from a rural environment. Families of origin were nuclear in 65% (*n* = 26), multigenerational in 5% (*n* = 2) and cohabiting unmarried couples in 17.5% (*n* = 7); family structure was not recorded for 12.5% (*n* = 5). Educational attainment was gymnasium in 10% (*n* = 4), high school in 30% (*n* = 12) and higher education in 55% (*n* = 22), and was not recorded for 5% (*n* = 2). All dyads were hospitalised in the context of delivery.

### 3.2. Pre-Delivery Haematology and Inflammatory Status

**Figure 2:**
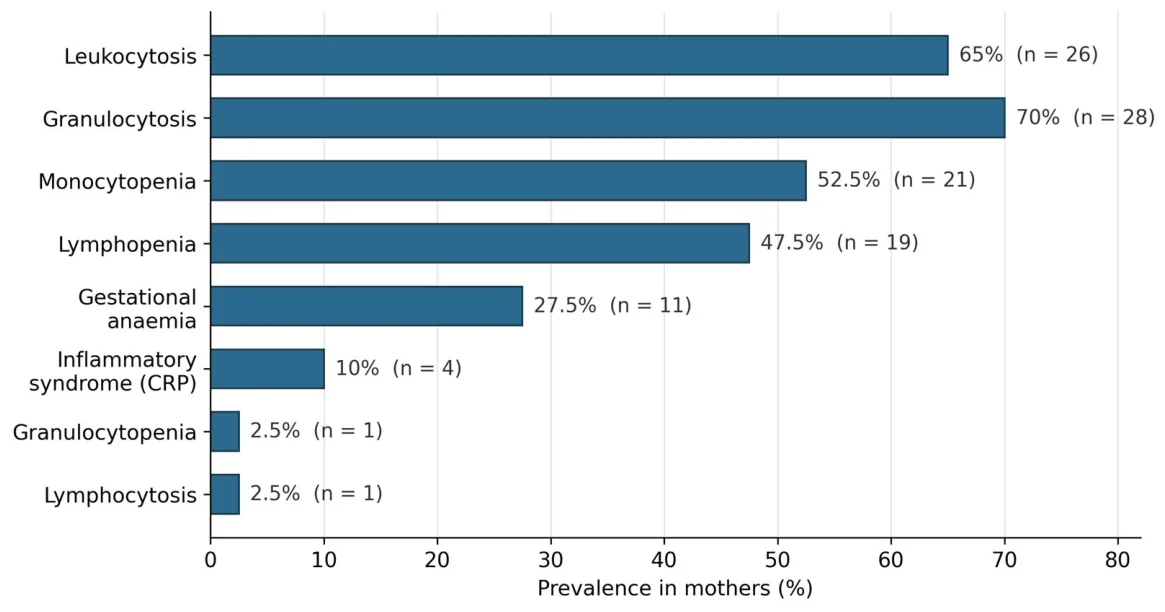
Prevalence of pre-delivery haematological and inflammatory findings in mothers (*n* = 40). CRP, C-reactive protein.

The pre-delivery complete blood count showed leukocytosis (9.81–16 × 10^3^/µL) in 65% (*n* = 26) of mothers, with granulocytosis (70.5–91.3%) in 70% (*n* = 28) and, less frequently, granulocytopenia (*n* = 1) or lymphocytosis (*n* = 1); lymphopenia was present in 47.5% (*n* = 19) and monocytopenia in 52.5% (*n* = 21) (Figure 2). Gestational anaemia affected 27.5% (*n* = 11) of mothers (haemoglobin 8.87–11.45 g/dL; haematocrit 26.3–34.3%); with ferritin within normal limits, the anaemia was further characterised using erythrocyte and platelet indices (Figure 2; Table 1).

Median maternal CRP was 6.65 mg/L (IQR 5.275–9.075; W = 0.923, *p* = 0.015), with an inflammatory or infectious syndrome—defined as CRP above 12 mg/L—in 10.8% (4 of the 37 mothers with CRP available). Neonatal CRP was lower (MED 2.405 mg/L, IQR 1.278–4.52; W = 0.672, *p* < 0.001), and the observed range was wider in newborns (0.56–24.52 mg/L) than in mothers (5.2–16.0 mg/L).

### 3.3. Serum eNOS on the First Postpartum Day

**Figure 3:**
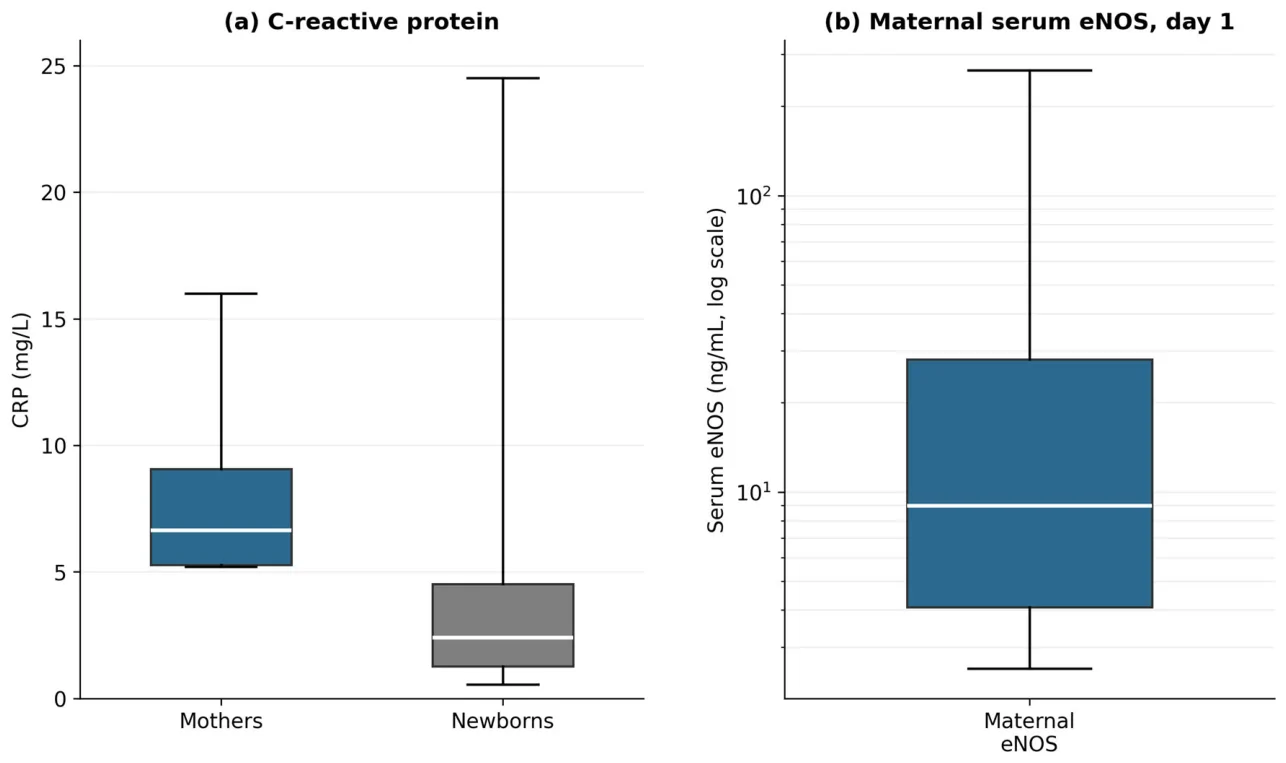
Early postpartum serum analyses: (**a**) C-reactive protein in mothers and newborns (box: median and IQR; whiskers: range); (**b**) maternal serum eNOS on postpartum day 1 (log scale; median 8.997 ng/mL, IQR 4.091–27.985; W = 0.61, *p* < 0.001).

Maternal serum eNOS levels, extremely skewed to the right and not normally distributed (W = 0.61, *p* < 0.001), had a median value of 8.997 ng/mL (IQR 4.091–27.985) and a wide range (2.537–264.737 ng/mL); the corresponding mean ± SD was 31.237 ± 51.607 ng/mL, reflecting the influence of a single extreme value (264.7 ng/mL) (Figure 3). Given this skew, the median was used as the primary summary; the extreme observation was retained in the primary rank-based analysis, which is robust to its magnitude, and its influence on the secondary linear regression models is addressed in Section 3.6.

### 3.4. Correlates of Serum eNOS

**Figure 4:**
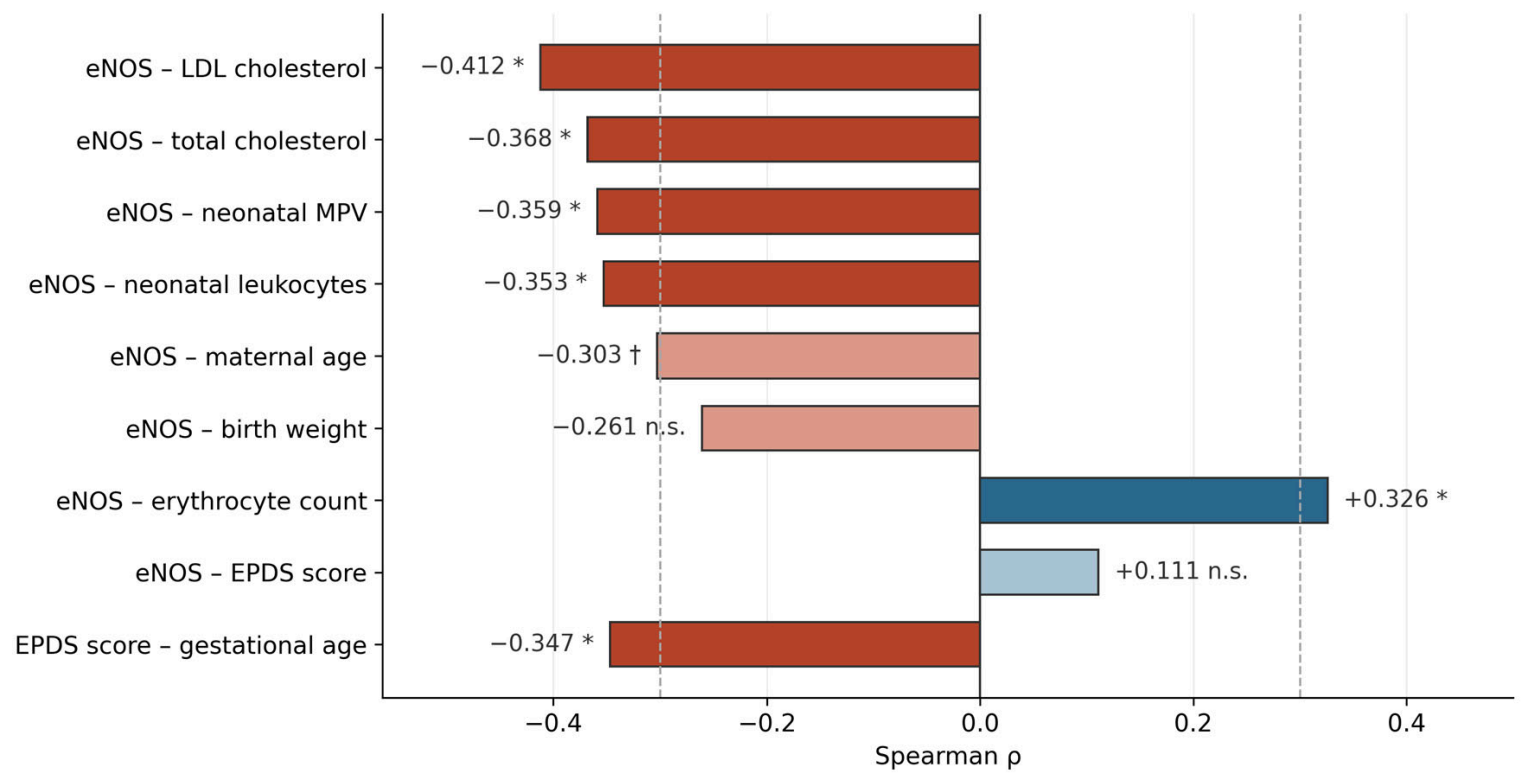
Exploratory Spearman correlations of maternal serum eNOS (and of EPDS score with gestational age); the number of participants contributing to each pair is given in Supplementary Table S2. * *p* < 0.05; † 0.05 ≤ *p* < 0.10; n.s. not significant. *p*-values are unadjusted for multiple comparisons; dashed lines mark |ρ| = 0.30. EPDS, Edinburgh Postnatal Depression Scale; MPV, mean platelet volume.

Serum eNOS correlated inversely and moderately with maternal age, although this association fell short of significance (ρ = −0.303, *p* = 0.061, *n* = 39), and was not associated with neonatal birth weight (ρ = −0.261, *p* = 0.104, *n* = 40) (Figure 4). Among maternal redox-relevant markers, eNOS correlated positively with the erythrocyte count (ρ = 0.326, *p* = 0.045, *n* = 38) and negatively with total cholesterol (ρ = −0.368, *p* = 0.027, *n* = 36) and low-density lipoprotein (LDL) cholesterol (ρ = −0.412, *p* = 0.013, *n* = 36). Turning to neonatal parameters on day 1, maternal eNOS varied inversely with the newborn’s white blood cell count (ρ = −0.353, *p* = 0.025, *n* = 40) and with neonatal mean platelet volume (ρ = −0.359, *p* = 0.023, *n* = 40). The intensity of depressive symptoms was not associated with maternal eNOS (ρ = 0.111, *p* = 0.512, *n* = 37); however, the early postpartum maternal EPDS score did vary inversely with the infants’ gestational age at birth (ρ = −0.347, *p* = 0.035, *n* = 37).

All correlations are unadjusted for multiple testing and are exploratory (Figure 4; the underlying coefficients and exact *p*-values are provided in Supplementary Table S2).

### 3.5. Postnatal Depression Screening

The median EPDS score was 7 (IQR 4–9; W = 0.936, *p* = 0.030). Screening identified possible depression (EPDS 9–11) in 20% of mothers (*n* = 8) and probable depression (EPDS ≥ 14) in 5% (*n* = 2); the remaining 30 mothers (75%) scored below 9 or in the intermediate 12–13 band. The Romanian-language EPDS-R, administered to a subgroup of 29, gave a concordant central tendency (median 6, IQR 3–9.5) and classified the same participants in the same broad bands. Only total scores were available for the present analysis, so agreement between the two versions is reported at the distributional level and no internal-consistency coefficient is presented; this is stated among the limitations (Section 4.6). Age-stratified EPDS scores are summarised in Table 2.

### 3.6. Regression Models and Sensitivity Analysis

**Figure 5:**
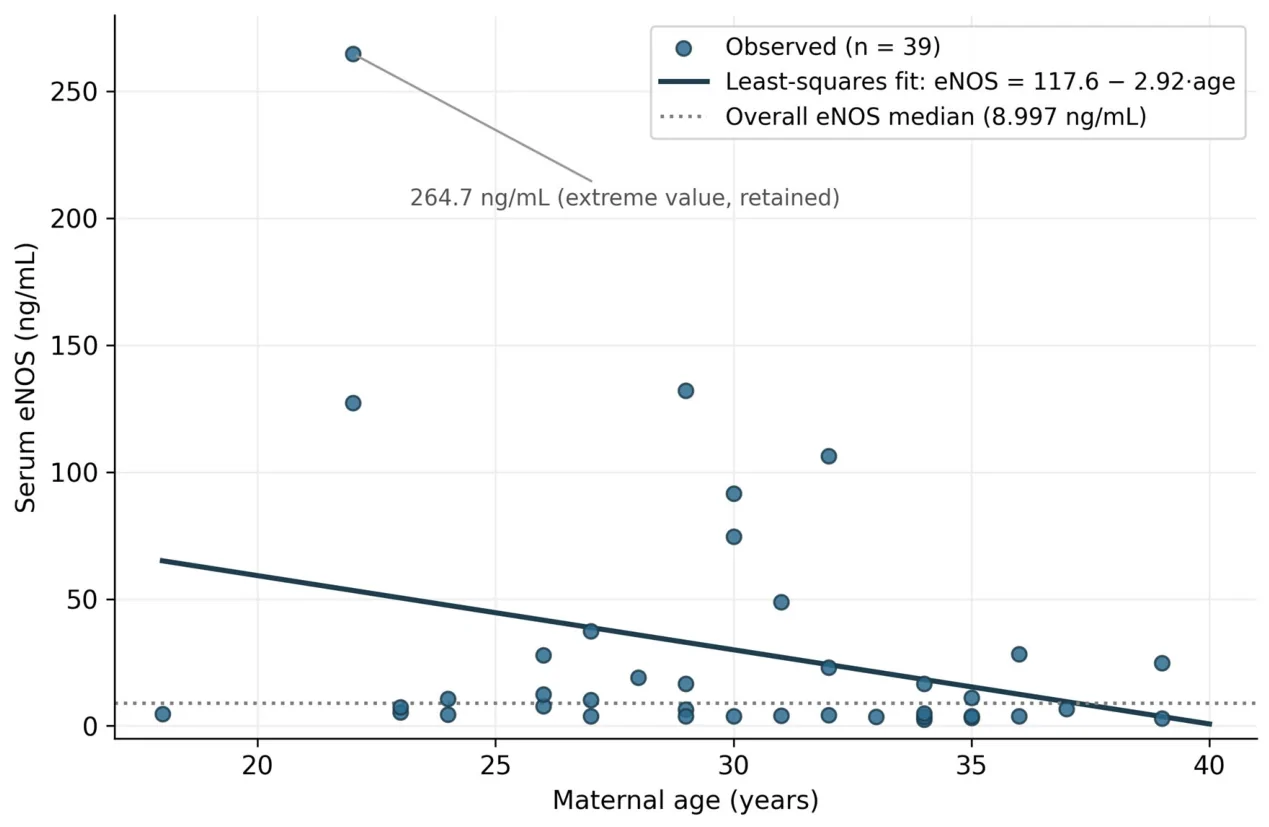
Simple linear regression of maternal serum eNOS on maternal age (*R*^2^ = 0.081; F(1,37) = 3.27; β = −2.92; *p* = 0.079; Spearman ρ = −0.303, *p* = 0.061; *n* = 39). The least-squares regression line is shown with the observed data; the linear model is secondary to the rank-based analysis and is sensitive to an outlier (see Section 3.6).

In the complementary simple linear regression (secondary analysis), the inverse association between maternal age and serum eNOS did not reach significance (*R*^2^ = 0.081; adjusted *R*^2^ = 0.056; F(1,37) = 3.27; β = −2.92; *p* = 0.079); the linear model accounted for 8.1% of the variability in eNOS (Figure 5), with a posthoc power of only 44% (*f*^2^ = 0.09). This *R*^2^ describes the fit of the linear model alone and is not interchangeable with the rank-based Spearman coefficient (ρ = −0.303, *p* = 0.061), which constitutes the primary analysis. The least-squares estimates are also sensitive to the single extreme eNOS value (264.737 ng/mL), whereas the rank-based analysis is unaffected by its magnitude. The slope should not be read as a dose–response estimate: extrapolated across the observed age range on the untransformed scale, it would predict negative concentrations—a further sign that a linear model fits these skewed data poorly; on the log scale, the association is weaker still (*r* = −0.235, *p* = 0.149). Two multivariable models were also fitted: (i) maternal age plus erythrocyte count (*R*^2^ = 0.139; adjusted *R*^2^ = 0.088; F(2,34) = 2.74; *p* = 0.079) and (ii) total cholesterol plus LDL cholesterol plus creatinine (*R*^2^ = 0.102; adjusted *R*^2^ = 0.017; F(3,32) = 1.21; *p* = 0.324). Neither was significant and no individual predictor reached significance (all *p* > 0.05). Given the sensitivity analysis in Section 2.7, which shows that only large effects (*R*^2^ ≈ 0.22–0.26) were detectable at 80% power, this pattern is uninformative rather than evidence of no association; it is also consistent with variance shared among correlated predictors, since total and LDL cholesterol are collinear by construction (Table 3). Because the extreme value exerts high leverage on least-squares estimation and the sample is small, coefficient-level estimates for the multivariable models are reported only in Supplementary Table S4, and these models are regarded as strictly exploratory. Accordingly, no variable is designated an independent predictor of eNOS.

### 3.7. Prevalence Odds and Age-Related Odds Ratios

Prevalences are reported with exact binomial 95% confidence intervals and the corresponding odds, each computed on the participants for whom the defining measurement was available. A pre-delivery inflammatory or infectious syndrome, defined as C-reactive protein above 12 mg/L, was present in 10.8% (4/37; 95% CI 3.0–25.4; odds 0.121:1). Gestational anaemia, by the operational rule of Section 2.6, affected 24.3% (9/37; 95% CI 11.8–41.2; odds 0.321:1); applying the WHO threshold of haemoglobin below 11.0 g/dL to the same 37 participants gives 18.9% (7/37; 95% CI 8.0–35.2; odds 0.233:1), and the two definitions agree on seven of the nine cases. Neonatal early infection or inflammation, defined as C-reactive protein above 10 mg/L, affected 16.7% (6/36; 95% CI 6.4–32.8; odds 0.200:1). Screening was positive for depressive symptoms, defined as an EPDS score of 9 or above, in 25.0% (10/40; 95% CI 12.7–41.2; odds 0.333:1). Advancing maternal age was associated with higher odds of the newborn requiring oxygen therapy, although the estimate was imprecise and only marginally significant (OR = 7.6; 95% CI 0.746–77.434; z = 1.712; *p* = 0.087). Maternal age was not associated with gestational anaemia (OR = 1.333; 95% CI 0.191–9.312; *p* = 0.772), inflammatory syndrome (OR = 3.0; 95% CI 0.168–53.713; *p* = 0.455) or screen-positive depression (OR = 1.56; 95% CI 0.125–19.597; *p* = 0.729). The very wide confidence intervals, several of which span more than two orders of magnitude, mean that these odds-ratio analyses carry essentially no inferential weight and are presented for completeness only.

## 4. Discussion

### 4.1. Principal Findings

In this exploratory cross-sectional study of the first postpartum day, three observations stand out. First, circulating eNOS protein tracked inversely with total and LDL cholesterol and positively with erythrocyte count, a pattern coherent with the redox biology of the enzyme and the only set of associations to reach conventional significance. Second, eNOS was inversely associated with maternal age with a coefficient of comparable magnitude (ρ = −0.303), but this fell short of significance (*p* = 0.061) and the secondary linear model was likewise non-significant and underpowered (*R*^2^ = 0.081, *p* = 0.079, power 44%); maternal age is therefore best described as a possible correlate awaiting confirmation, not as a predictor. Third, eNOS was not associated with the intensity of depressive symptoms. Because the study had only about 10% power to detect a correlation of the magnitude observed (ρ = 0.111), this third result is an absence of evidence rather than evidence of absence, and it neither supports nor excludes the biologically plausible link it was designed to probe.

### 4.2. eNOS, Maternal Age and Redox Balance

The inverse age–eNOS association, though not statistically significant here, is biologically coherent and worth stating as a hypothesis. Ageing is accompanied by increased vascular oxidative stress, which lowers eNOS expression and promotes uncoupling, both of which diminish NO-generating capacity and eNOS-derived signalling; in replicative endothelial senescence this proceeds through a rise in the BH2/BH4 ratio and a fall in BH4 availability [58], and we will come back with some important information. The parallel inverse associations with total and LDL cholesterol are consistent with the same framework: oxidised LDL impairs eNOS activity and NO bioavailability and promotes endothelial dysfunction, a redox-mediated process central to atherogenesis [59]. That the linear model including maternal age explained only a small share of the variability in eNOS, and did not reach significance (*R*^2^ = 0.081; Section 3.6), underscores that circulating eNOS is multifactorial and, in the early puerperium, is likely also shaped by the dynamic haemodynamic and hormonal changes in delivery. The positive association with erythrocyte count is notable because erythrocytes themselves express a functional eNOS that contributes to circulating NO metabolites and blood-pressure regulation [60,61]; our data are compatible with a red-cell contribution to the circulating eNOS pool, though the assay does not distinguish cellular origin. We emphasise that these mechanistic interpretations rest on external experimental evidence and are not directly demonstrated by our data, which quantified total immunoreactive eNOS protein rather than enzyme activity, phosphorylation status or coupling state (Section 2.4 and Section 4.6).

It is worth setting out explicitly the mechanism that would account for the age association, together with the measurements that would be required to test it. Tetrahydrobiopterin (BH4) is the obligatory cofactor for coupled eNOS catalysis. When oxidative stress oxidises BH4 to BH2 and the BH4/BH2 ratio falls, the enzyme uncouples: electron flow is diverted to molecular oxygen, so eNOS produces superoxide rather than NO. Superoxide and residual NO combine to form peroxynitrite, which oxidises BH4 further and nitrates tyrosine residues, giving a self-amplifying cycle in which 3-nitrotyrosine accumulates as a footprint of nitrosative stress [17,18,58]. Two additional routes converge on the same endpoint and are relevant to the lipid associations reported here: oxidised LDL impairs eNOS coupling and NO bioavailability [59], and asymmetric dimethylarginine (ADMA), an endogenous competitive inhibitor of eNOS that rises with age and with cardiometabolic risk, reduces NO output independently of enzyme abundance. Replicative endothelial senescence reproduces this sequence in vitro, with a raised BH2/BH4 ratio, reduced BH4 availability and lower eNOS expression [58]. This chain is biologically plausible and would connect increasing maternal age to reduced NO-generating capacity, but we must be clear that none of its steps was measured in the present study. We quantified total immunoreactive eNOS protein and nothing else: no BH4 or BH2, no BH4/BH2 ratio, no oxidised LDL, no 3-nitrotyrosine, no ADMA, and no nitrite or nitrate. The mechanism is therefore offered as the hypothesis that our observation generates, not as an inference our data support; Section 4.7 sets out the measurements needed to test it.

### 4.3. Comparison with Reported eNOS Concentrations

Reported circulating eNOS concentrations vary widely with matrix (serum versus plasma), assay and population, which makes cross-study comparison difficult. Plasma eNOS is lower in non-pregnant women and in hypertensive disorders of pregnancy than in healthy pregnancy, and correlates positively with haemoglobin and birth weight, and inversely with proteinuria [25,26], which differs from hypertensive pathology other than that from pregnancy [62]. In non-pregnant adults, plasma eNOS measured by immunoassay has been reported in the low single-digit ng/mL range, for instance 4.05 ± 1.44 ng/mL in controls and 3.00 ± 0.94 ng/mL after acute myocardial infarction [62]. Our median serum value of about 9 ng/mL is therefore higher than these plasma reports, and the single extreme value of 264.737 ng/mL is a clear outlier. Several explanations are compatible with the data and we cannot distinguish between them: the serum rather than plasma matrix, release of platelet- and erythrocyte-derived eNOS during clotting, lot-specific assay calibration, or a genuine biological subgroup. Serum is the most likely single contributor, because clot formation lyses platelets and releases their eNOS into the measured compartment, which plasma collection avoids. This reinforces the need to report full assay-validation metrics, to pre-specify outlier handling, and to standardise the matrix in future work; until that is done, absolute concentrations from different studies should not be compared directly. The inverse association with birth weight was only marginal here and runs opposite to the positive plasma-eNOS-birth-weight association reported in hypertensive pregnancy [25], so it should be regarded as hypothesis-generating. Fundamentally, the NO/eNOS pathway is vasoprotective and antiatherogenic [63].

### 4.4. eNOS and Postnatal Depression

We found no evidence of an association between serum eNOS protein and EPDS score (ρ = 0.111, *p* = 0.512). This must not be read as a demonstration that no association exists: the posthoc power to detect a correlation of that size was approximately 10%, and the study could reliably detect only |ρ| ≥ 0.45 (Section 2.7). NO participates in neurotransmitter release, neuronal excitability and synaptic plasticity, and eNOS at the neurovascular unit can support rapid, activity-coupled NO production [64], so the hypothesis remains open and requires an adequately powered test. The screening results were as expected, with possible depression in 20% and probable depression in 5% of mothers, values falling within internationally reported ranges [33,65]. EPDS scores varied inversely with gestational age at birth, which is consistent with the greater vulnerability described after earlier or premature births. Peripartum depression arises from a convergence of rapid hormonal change, neuroendocrine dysregulation and psychosocial stressors rather than from any single biomarker [34,35,40,66]. Endothelial NO also contributes to the preservation of cognitive health [66], and progesterone, which falls abruptly after delivery, is itself a positive regulator of eNOS expression [67], so the puerperal hormonal transition may plausibly act on vascular and affective pathways in parallel. Given the hormonal implications (and not just those), whatever the mechanism, systematic early screening remains essential because a substantial proportion of cases would otherwise go undetected [37,67].

### 4.5. Maternal–Neonatal Associations

Maternal eNOS correlated inversely with neonatal leukocyte count and MPV, and older maternal age was associated (imprecisely) with a greater need for neonatal oxygen therapy. Given the vasoactive and immunomodulatory roles of NO in the transitional neonatal circulation and its therapeutic use as inhaled NO [68], these maternal–neonatal signals are plausible but exploratory. NO produced by eNOS contributes to lactation physiology and early gastrointestinal protection of the newborn through nitrite in colostrum and breast milk [31,32,69], linking maternal NO to the baby’s well-being.

### 4.6. Strengths and Limitations

Strengths include a well-defined single-day sampling window, standardised pre-analytical handling, triplicate ELISA measurement, and the integration of biochemical, haematological and validated mental-health screening in the same dyads. The limitations are substantial and constrain what may be concluded. (i) The sample is small and drawn from a single centre (*n* = 40). Power was sufficient only for moderate-to-strong correlations (|ρ| ≥ 0.45) and for large effects in regression (*R*^2^ ≈ 0.22–0.25); confidence intervals are wide; and adjustment for important confounders was not possible, including pre-gestational and gestational body mass index, mode of delivery, smoking, medication, diet and gestational pathology. (ii) All participants were recruited at one Romanian maternity unit over a single month, so the findings describe this population and should not be extrapolated to postpartum women in other settings, ethnic groups or healthcare systems without replication. (iii) The cross-sectional design, with one measurement point, establishes neither temporality nor causality. (iv) Correlations were not corrected for multiple comparisons and are exploratory. (v) The assay quantifies total immunoreactive eNOS protein. It does not distinguish phosphorylated (Ser1177) from non-phosphorylated enzyme or dimeric from monomeric forms, and protein concentration is not a measure of enzymatic activity; no direct redox biomarker was measured, so neither eNOS coupling nor actual NO output is demonstrated by these data. Specifically, we did not measure BH4, BH2 or the BH4/BH2 ratio, oxidised LDL, 3-nitrotyrosine, asymmetric dimethylarginine, F2-isoprostanes or serum nitrite and nitrate, and we did not genotype NOS3 (for example rs1799983/Glu298Asp, rs2070744/−786T>C). (vi) The serum matrix and a single extreme value complicate comparison with the plasma literature. (vii) The LOD and LLOQ were taken from the manufacturer’s validation and were not re-verified in-house in this serum matrix, although all measured concentrations exceeded the LLOQ more than six-fold (Section 2.4). (viii) The Romanian EPDS-R was validated in an antenatal population, and its optimal cut-point was derived in pregnancy rather than in the puerperium, so the subgroup comparison is a consistency check rather than a validation of postnatal performance; only total scores were available for analysis, so no internal-consistency coefficient could be computed for the EPDS-R. (ix) Gestational anaemia was classified by an operational rule (haemoglobin < 11.5 g/dL together with haematocrit < 35%) whose thresholds lie above those in WHO [49] and UK [50] guidance, and which requires both criteria rather than either, so the prevalence reported here is not comparable with guideline-based figures (Section 2.6). The sensitivity analysis requested by this comparison is reported in Section 3.7: applying the WHO threshold to the same 37 participants gives 18.9% rather than 24.3%, and the two definitions agree on seven of the nine cases. (x) Nine haematological values fell outside physiological limits. Three were traced to unambiguous cell duplication during data entry and six had no identifiable source; all nine were excluded from Table 1 and reported as missing, which materially changed the affected summaries (maternal RDW-CV, for example, falls from 18.84 ± 22.81 to 13.89 ± 2.06). The underlying records should be re-checked, and the possibility that other, less conspicuous transcription errors remain cannot be excluded. These limitations define the agenda for future work.

### 4.7. Future Directions

The priority is to move from an observed association to a tested mechanism, and three steps are needed in this order. First, adequately powered, longitudinal, multi-centre cohorts should sample across pregnancy and the puerperium, with sample sizes derived from the effects observed here rather than from convenience; detecting a correlation of ρ ≈ 0.30 at 80% power requires roughly 85 participants, and a mediation analysis requires considerably more. Second, direct redox phenotyping in the same samples, measuring BH4 and BH2 with the BH4/BH2 ratio as the index of cofactor sufficiency, oxidised LDL, 3-nitrotyrosine as a footprint of peroxynitrite-mediated protein nitration, asymmetric dimethylarginine as the endogenous eNOS inhibitor, F2-isoprostanes, and nitrite and nitrate as the downstream index of NO output. These should be paired with direct markers of eNOS functionality, namely phosphorylated eNOS at Ser1177 and the eNOS dimer-to-monomer ratio, so that protein abundance, coupling state and NO production can be distinguished rather than conflated. Third, and critically for the mechanistic question this study raises but cannot answer, the analysis should be structured to test the proposed causal chain rather than its endpoints alone: whether maternal age predicts a fall in the BH4/BH2 ratio and a rise in ADMA and 3-nitrotyrosine, and whether those changes in turn predict lower eNOS-derived NO output, with formal mediation analysis and adjustment for adiposity, parity and mode of delivery. NOS3 genotyping (rs1799983, rs2070744) would clarify how much of the between-individual variation is constitutive, and measuring eNOS in additional compartments such as breast milk and cord blood would probe maternal-foetal transfer. Because the pathway is redox-dependent, dietary nitrate, L-arginine and antioxidant supplementation such as N-acetylcysteine, which reverses eNOS-associated endothelial epigenetic programming in experimental growth restriction [21,22,70], merit controlled evaluation, though no clinical recommendation follows from the present data. From a maternal public-health perspective, pairing a compact biomarker panel with routine EPDS screening could support earlier identification of vascular and mental-health risk in the puerperium, but only once the underlying associations have been confirmed prospectively.

## 5. Conclusions

On the first postpartum day, lower circulating eNOS protein was accompanied by a less favourable lipid profile, and higher eNOS was accompanied by a higher erythrocyte count; the inverse association with maternal age was of similar magnitude but did not reach significance. These patterns are consistent with an age- and redox-related decline in NO-generating capacity, but consistency is not demonstration: because the assay quantifies total eNOS protein, the findings concern circulating eNOS abundance rather than enzymatic activity, coupling state or NO output, and no redox biomarker was measured. The multivariable models were not significant and were powered to detect only large effects, so no variable can be described as an independent predictor. Circulating eNOS was unrelated to the intensity of early depressive symptoms, a result that reflects an absence of evidence rather than evidence of absence, given approximately 10% power at the observed effect size. These are exploratory findings from a single Romanian centre and generate hypotheses rather than conclusions. They require confirmation in larger, longitudinal and multi-centre cohorts incorporating direct redox biomarkers, markers of eNOS activity such as phosphorylated eNOS, the dimer-to-monomer ratio and nitrite/nitrate, and NOS3 genotyping, before any clinical inference is drawn.

## Figures and Tables

**Table 1:** Erythrocyte and platelet indices in mothers (pre-delivery) and newborns (postpartum day 1), with reference intervals.

Group/Statistic	MCV (fL)	MCH (pg)	MCHC (g/dL)	RDW-CV (%)	PDW (%)	MPV (fL)
Mothers—range	75–94.7	24.2–32.5	30.4–35.3	12.1–21.1	15.7–18.6	7.7–11.5
Mothers—M ± SD	86.63 ± 5.64	28.77 ± 2.22	33.05 ± 1.30	13.89 ± 2.06	17.04 ± 0.65	8.74 ± 0.95
Mothers MED (IQR)	89.10 (8.60)	29.16 (3.38)	33.10 (2.25)	13.20 (1.55)	17.10 (0.50)	8.60 (0.90)
Reference (women)	79–98	27–32	32–36	11.6–14.0	9.7–17.0	7.4–10.4
Newborns range	94.2–110.7	31.4–38.0	32.3–37.7	13.7–19.5	8.6–18.3	7.3–16.9
Newborns M ± SD	102.78 ± 3.78	35.56 ± 1.43	34.62 ± 1.08	16.91 ± 1.56	11.12 ± 2.79	9.44 ± 1.51
Newborns MED (IQR)	102.95 (4.92)	35.65 (2.23)	34.75 (1.02)	16.90 (1.98)	10.20 (1.65)	9.40 (1.05)
Reference (newborn)	98–114	32–40	32–36	15.5–20	analyser-dependent	7.9–11.0

M ± SD is reported when Shapiro–Wilk *p* > 0.05 and MED (IQR) when *p* < 0.05. The value in parentheses after each median is the IQR width (Q3 − Q1). Adult female reference intervals are those of an automated haematology analyser in a healthy adult population [46,47]; neonatal intervals are for term newborns in the first 24–48 h of life [55,56,57]. Neonatal PDW and MPV intervals are strongly instrument- and sampling-site dependent and are not transferable between analysers, so no single interval is quoted for neonatal PDW. PDW is reported as the coefficient-of-variation form (PDW-CV, %); the CV (%) and SD (fL) forms are not interchangeable. Nine values were excluded from these summaries as non-physiological. Three were unambiguous cell duplications in the source records and are reported as missing: haemoglobin 39.3 g/dL duplicated from the haematocrit of the same participant, and MCH values of 9.5 pg and 93.2 pg duplicated from that participant’s haemoglobin and MCV respectively. Six further values had no identifiable source and were also excluded: maternal MCV 236 fL, maternal RDW-CV 118% and 355%, and the maternal PDW (107%) and MPV (210 fL) of a single participant, together with a neonatal MPV of 0.9 fL.MCV, mean corpuscular volume; MCH, mean corpuscular haemoglobin; MCHC, mean corpuscular haemoglobin concentration; RDW-CV, red cell distribution width; PDW, platelet distribution width; MPV, mean platelet volume; M—mean; SD—standard deviation; MED—median; IQR—interquartile range.

**Table 2:** Maternal serum eNOS and EPDS by maternal age group.

Variable	18–20 y (*n* = 1)	21–30 y (*n* = 20)	31–40 y (*n* = 18)
eNOS (ng/mL), MED (IQR) (range)	4.795	11.592 (40.466) (3.766–264.737)	4.656 (17.787) (2.537–106.250)
EPDS score, MED (IQR) (range)	4	6 (3) (0–21)	7 (3) (1–10)
Possible PPD (EPDS 9–11), *n*	0	5	3
Probable PPD (EPDS ≥ 14), *n*	0	2	0

The value in parentheses after each median is the IQR width (Q3 − Q1). PPD, postnatal depression; EPDS, Edinburgh Postnatal Depression Scale; MED, median; IQR, interquartile range. The eNOS row is computed from the study records; the three bands cover 39 of the 40 mothers because maternal age was not recorded for one participant (serum eNOS 74.868 ng/mL), who is therefore not assignable to a band.

**Table 3:** Linear regression models predicting maternal serum eNOS level (ng/mL).

Model	*R^2^*	Adjusted *R*^2^	β (Unstandardised)	*p*
Simple: maternal age	0.081	0.056	−2.92	0.079
(i) maternal age + erythrocyte count	0.139	0.088	n.s. predictors	0.079
(ii) total chol. + LDL chol. + creatinine	0.102	0.017	n.s. predictors	0.324

Simple model: F(1,37) = 3.27; effect size *f*^2^ = 0.09; posthoc power 44% at α = 0.05. n.s., no individually significant predictor (all *p* > 0.05). For the multivariable models a sensitivity analysis is more informative than observed power: with α = 0.05, the smallest effect detectable at 80% power is *f*^2^ = 0.29 (*R*^2^ ≈ 0.22) for the two-predictor model and *f*^2^ = 0.34 (*R*^2^ ≈ 0.26) for the three-predictor model, so only large effects were within reach and non-significance is uninformative. Coefficient-level estimates for the multivariable models are given in Supplementary Table S4 and are not reproduced here, because no predictor reached significance and the small, outlier-sensitive sample precludes stable estimation. Total and LDL cholesterol are collinear by construction, so model (ii) cannot separate their contributions. chol., cholesterol; LDL, low-density lipoprotein.

## Data Availability

The STROBE checklist is deposited in Zenodo (DOI: 10.5281/zenodo.21511196). The anonymised individual-level dataset and the analysis script are available from the corresponding author on reasonable request, subject to approval by the institutional ethics committee, because perinatal mental-health data could permit re-identification of participants.

## Supplementary Materials

The following supporting information can be downloaded at: https://www.mdpi.com/article/10.3390/antiox15091176/s1.

## Abbreviations

The following abbreviations are used in this manuscript:

BH4	tetrahydrobiopterin
BH2	dihydrobiopterin
ADMA	asymmetric dimethylarginine
BMI	body mass index
CI	confidence interval
CRP	C-reactive protein
ELISA	enzyme-linked immunosorbent assay
CV	coefficient of variation
eNOS	endothelial nitric oxide synthase
EPDS	Edinburgh Postnatal Depression Scale
EPDS-R	Romanian-language version of the EPDS
iNOS	inducible nitric oxide synthase
IQR	interquartile range
LDL	low-density lipoprotein
LOD	limit of detection
LLOQ	lower limit of quantification
MCH	mean corpuscular haemoglobin
MCHC	mean corpuscular haemoglobin concentration
MCV	mean corpuscular volume
M	mean
MED	median
MPV	mean platelet volume
NO	nitric oxide
nNOS	neuronal nitric oxide synthase
NOS3	nitric oxide synthase 3
OR	odds ratio
RBC	red blood cell
PDW	platelet distribution width
PPD	postnatal depression
RDW-CV	red cell distribution width (coefficient of variation)
SD	standard deviation
STROBE	Strengthening the Reporting of Observational Studies in Epidemiology
