# Supplementary material for: Circulating Endothelial Nitric Oxide Synthase on the First Postpartum Day: An Exploratory Cross-Sectional Study of Its Association with Maternal Age, Redox-Relevant Haematological and Lipid Markers, and Postnatal Depression Screening
Source: Antioxidants (Basel). 2026 Sep 16;15(9):1176. doi: 10.3390/antiox15091176 (PMC13603924; doi:10.3390/antiox15091176)
Supplement: Supplementary file 1 [file antioxidants-15-01176-s001.zip › antioxidants-4535196-supplementary.pdf]

Table S1. STROBE Statement: Checklist of Items for Cross-Sectional Studies

Manuscript: "Circulating Endothelial Nitric Oxide Synthase on the First Postpartum Day: An Exploratory Cross-Sectional Study of Its Association with Maternal Age, Redox-Relevant Haematological and Lipid Markers, and Postnatal Depression Screening". Section/page references point to the main manuscript.

**Item**	**STROBE recommendation**	**Location in manuscript**
Title & abstract (1)	(a) Design in title/abstract; (b) structured summary of what was done/found	Title (states 'cross-sectional'); structured Abstract
Background/rationale (2)	Scientific background and rationale	Introduction, paras 1–6
Objectives (3)	Specific objectives/hypotheses	Introduction, final paragraph (objectives a–d)
Study design (4)	Key design elements early in paper	Abstract; Section 2.1
Setting (5)	Setting, locations, dates of recruitment/data collection	Section 2.1 (Timișoara; July 2022)
Participants (6)	Eligibility criteria; sources and selection methods	Section 2.2 (inclusion/exclusion bullets); Figure 1
Variables (7)	Outcomes, exposures, predictors, confounders, effect modifiers	Sections 2.3–2.6; confounders not collected noted in 4.6
Data sources/measurement (8)	Sources and methods of assessment; comparability	Sections 2.3 (sampling), 2.4 (ELISA), 2.5 (EPDS), 2.6 (indices)
Bias (9)	Efforts to address potential sources of bias	Sections 2.4 (triplicate, calibration), 2.7 (multiplicity, outlier); 4.6
Study size (10)	How study size was arrived at	Sections 2.2, 2.7 (sensitivity analysis: minimum detectable $\|\rho\| \approx 0.45$; minimum detectable $R^2 \approx 0.22–0.25$ for the multivariable models)
Quantitative variables (11)	Handling of quantitative variables/groupings	Section 2.7 (median (IQR) vs mean ± SD by Shapiro–Wilk); age groups Table 2
Statistical methods (12)	(a–e) all methods, subgroups, missing data, sensitivity analyses	Section 2.7 (Spearman primary; regression secondary with post-hoc power and sensitivity analysis; prevalence odds; unadjusted p; n per analysis; outlier handling)
Participants (13)	Numbers at each stage; reasons for non-participation	Section 3.1; Figure 1 (n=40; eNOS n=37; EPDS-R n=29)
Descriptive data (14)	Characteristics; missing data per variable	Section 3.1; Tables 1, 2; analysable n stated
Outcome data (15)	Numbers of outcome events/summary measures	Sections 3.2–3.7; Tables 1–3; Table S2
Main results (16)	(a) Estimates + 95% CI; (b) category boundaries; (c) absolute risk	Sections 3.4, 3.6, 3.7; Table 3; Table S2 (ρ, p and n per pair); prevalences with exact binomial 95% CI and odds in Section 3.7
Other analyses (17)	Subgroups, interactions, sensitivity analyses	Section 3.5 (age strata); 3.6 (outlier influence on linear models, flagged)
Key results (18)	Summary with reference to objectives	Section 4.1

Limitations (19)	Limitations, direction/magnitude of bias	Section 4.6 (items i–x)
Interpretation (20)	Cautious overall interpretation	Sections 4.2–4.5; Conclusions
Generalisability (21)	External validity	Abstract (Conclusions); Section 4.6, item (ii) (single centre, single Romanian population, one recruitment month)
Funding (22)	Funding source and role	Back matter: Funding statement

STROBE item 22 requires the funding source and the role of the funders to be stated. Adapted from von Elm, E.; Altman, D.G.; Egger, M.; Pocock, S.J.; Gotzsche, P.C.; Vandenbroucke, J.P. The Strengthening the Reporting of Observational Studies in Epidemiology (STROBE) statement: guidelines for reporting observational studies. Lancet 2007, 370, 1453-1457. https://doi.org/10.1016/S0140-6736(07)61602-X. Checklist available at www.strobe-statement.org.

Table S2. Exploratory Spearman correlations of maternal serum eNOS and of the EPDS score with selected parameters, unadjusted for multiple comparisons.

**Variable pair**	**Spearman ρ**	**p-value**	n
eNOS – maternal age	−0.303	0.061	39
eNOS – LDL cholesterol	−0.412	0.013	36
eNOS – total cholesterol	−0.368	0.027	36
eNOS – erythrocyte count	0.326	0.046	38
eNOS – neonatal MPV	−0.359	0.023	40
eNOS – neonatal leukocytes	−0.353	0.025	40
eNOS – birth weight	−0.261	0.104	40
eNOS – EPDS score	0.111	0.512	37
EPDS score – gestational age	−0.347	0.035	37

Spearman rank correlations, recomputed from the study records. *p*-values are two-sided and unadjusted for multiplicity; all associations are exploratory. Coefficients of |ρ| between 0.30 and 0.50 are described as moderate in the main text. The n column gives the participants contributing to each pair; it varies because the lipid panel, the erythrocyte count and maternal age were not available for every participant. The two EPDS rows are reported as originally analysed, because only total EPDS scores were available for the present analysis. EPDS, Edinburgh Postnatal Depression Scale; LDL, low-density lipoprotein; MPV, mean platelet volume; eNOS, endothelial nitric oxide synthase.

Table S3. Distribution of Edinburgh Postnatal Depression Scale scores across the screening bands defined in Section 2.5 of the main manuscript.

Screening band	EPDS score	Mothers, n	Mothers, %
Below the possible-depression threshold, or in the intermediate band	< 9 or 12–13	30	75.0
Possible depression	9–11	8	20.0
Probable depression	≥ 14	2	5.0
Total	0–30	40	100

EPDS bands follow Section 2.5 of the main manuscript. The counts for the 9–11 and ≥ 14 bands are those reported in Section 3.5; the first row combines the two remaining bands, because only the pooled count is available. The Romanian-language EPDS-R was administered to a subgroup of 29 mothers and gave a concordant central tendency (median 6, IQR 3–9.5, against a median of 7, IQR 4–9, for the EPDS in the full sample). Only total scores were available for the present analysis, so agreement between the two versions is reported at the distributional level and no internal-consistency coefficient is presented. The EPDS-R was validated in an antenatal population, so this comparison is a consistency check and not a validation of postnatal performance.

Table S4. Coefficient-level estimates for the simple and multivariable linear regression models of maternal serum eNOS (ng/mL) on postpartum day 1.

Predictor	β (unstandardised)	SE	95% CI	*t*	*p*
Simple model: maternal age (n = 39; R^2 = 0.081; adjusted R^2 = 0.056; F(1,37) = 3.27; p = 0.079)					
Intercept	117.61	49.04	18.23 to 216.98	2.398	0.022
Maternal age (years)	−2.921	1.615	−6.194 to 0.352	−1.808	0.079
Model (i): maternal age + erythrocyte count (n = 37; R^2 = 0.139; adjusted R^2 = 0.088; F(2,34) = 2.74; p = 0.079)					
Intercept	−38.93	117.80	−278.32 to 200.47	−0.330	0.743
Maternal age (years)	−2.980	1.648	−6.329 to 0.369	−1.808	0.079
Erythrocyte count (×10^6/µL)	37.94	25.50	−13.88 to 89.75	1.488	0.146
Model (ii): total cholesterol + LDL cholesterol + creatinine (n = 36; R^2 = 0.102; adjusted R^2 = 0.017; F(3,32) = 1.21; p = 0.324)					
Intercept	102.07	43.53	13.39 to 190.74	2.345	0.025
Total cholesterol (mg/dL)	−0.037	0.103	−0.248 to 0.173	−0.362	0.720
LDL cholesterol (mg/dL)	−0.346	0.228	−0.810 to 0.119	−1.515	0.139
Creatinine (mg/dL)	0.031	0.239	−0.455 to 0.517	0.129	0.898

Ordinary least-squares estimates computed from the study records; β is the change in serum eNOS (ng/mL) per unit of the predictor. All models are secondary and exploratory: the eNOS distribution is strongly right-skewed with one extreme value (264.7 ng/mL) that exerts high leverage, the sample is small, and only large effects ($R^2 \approx 0.22$–0.26) were detectable at 80% power (Section 2.7 of the main text). No predictor reached significance in the multivariable models and none should be read as an independent predictor of eNOS. Total and LDL cholesterol are collinear by construction, so model (ii) cannot separate their contributions. CI, confidence interval; LDL, low-density lipoprotein; SE, standard error.
